# The glucose-to-potassium ratio: a predictor of poor functional outcomes in stroke patients receiving thrombolytic therapy

**DOI:** 10.3389/fneur.2025.1581747

**Published:** 2025-06-05

**Authors:** Dong Zhang, Ruinan Ma, Xiaoyan Qin, Zhizhang Li, Xiaoguang Zhang, Ying Ding, Yunqi Hu, Yunhua Yue

**Affiliations:** ^1^Department of Neurology, Yangpu Hospital, School of Medicine, Tongji University, Shanghai, China; ^2^Department of Geriatrics, Yangpu Hospital, School of Medicine, Tongji University, Shanghai, China

**Keywords:** intravenous thrombolysis, GPR, functional outcome, acute ischemic stroke, biomarkers

## Abstract

**Background:**

The glucose-to-potassium ratio has shown promise as a biomarker in neurological disorders, but its prognostic value in acute ischemic stroke (AIS) after intravenous thrombolysis (IVT) continues to be uncertain. The study explores the relationship between admission GPR and 90-day functional outcomes in AIS patients undergoing IVT treatment.

**Methods:**

A retrospective analysis included 649 AIS patients undergoing IVT between May 2016 and December 2023. Baseline clinical, laboratory, and imaging data were analyzed. GPR was calculated from serum glucose and potassium levels at admission. A modified Rankin Scale score of 3 to 6 at 90 days was used to define poor functional outcomes. Logistic regression and restricted cubic splines assessed the GPR-outcome relationship, adjusting for confounders. Receiver operating characteristic (ROC) analysis evaluated GPR’s predictive value.

**Results:**

Among 649 patients, 174 (26.8%) had poor outcomes. Median GPR was significantly higher in these patients (2.14 vs. 1.88, *p* < 0.001). Higher GPR independently predicted negative consequences (OR, 1.821; 95% CI, 1.340–2.473, *p* < 0.001). Subgroup analysis indicated a stronger association in non-diabetic patients. ROC analysis demonstrated an area under the curve (AUC) of 0.631 (95% CI, 0.585–0.677, *p* < 0.001) for GPR in predicting poor functional outcomes.

**Conclusion:**

High GPR levels are independently linked to unfavorable 90-day functional outcomes in AIS patients who received IVT, suggesting its potential as a prognostic biomarker. Further studies are warranted to validate these findings.

## Introduction

Ischemic stroke has become the second most common cause of death and the third largest factor in global disability. Among those who survive cerebrovascular disease, over 70% experience varying degrees of work-related impairment, contributing to a growing global socioeconomic burden (1–3). Statistical data indicate that approximately 2 million individuals in China are affected annually, with around 8 million individuals currently living with the condition (4). The advent of intravenous thrombolysis (IVT) has revolutionized the treatment of acute ischemic stroke (AIS), yet the functional outcomes of patients undergoing this therapy remain highly variable. Identifying reliable predictors of prognosis following IVT is crucial for guiding treatment decisions and optimizing outcomes (5).

Serum biomarkers have emerged as important tools in predicting stroke outcomes, as they reflect underlying pathophysiological processes. Among these, glucose and potassium are critical components of cellular metabolism and ionic homeostasis, respectively. Hyperglycemia has long been associated with poor outcomes in AIS, possibly due to increased oxidative stress, inflammation, and blood–brain barrier disruption (6, 7). Similarly, hypokalemia may exacerbate neuronal injury through ionic imbalances and impaired cellular repair mechanisms (8).

As a novel biomarker, the glucose to potassium ratio (GPR) has been proposed, incorporating the prognostic aspects of both glucose and potassium levels. Prior investigations have revealed that elevated GPR correlates with adverse results in various neurological diseases, including traumatic brain injury, intracranial hemorrhage, and ischemic stroke treated with endovascular thrombectomy (5, 6). However, its role in predicting functional outcomes after IVT in AIS has not been determined. Consequently, this investigation focused on examining how GPR correlates with functional outcomes at the 90-day mark following IVT through a retrospective study.

## Methods and materials

### Study participants and design

This study is a retrospective analysis involving patients with acute ischemic stroke who underwent IVT at Yangpu Hospital of Tongji University School of Medicine, between May 2016 and December 2023. The inclusion of patients was determined by the following criteria: (1) 18 years old or above; (2) confirmation of cerebral infarction via imaging; and (3) availability of data necessary for the calculation of the GPR. To ensure consistency among study participants, we excluded patients who were treated with a combination of intravenous alteplase and endovascular thrombectomy (bridging therapy), those with renal failure, those with an estimated glomerular filtration rate (eGFR) of less than 60 mL/min/1.73 m^2^, those undergoing hemodialysis or peritoneal dialysis, and those lacking complete medical history, imaging reports, laboratory results, and National Institutes of Health Stroke Scale (NIHSS) scores. The Heidelberg Bleeding Classification was used to diagnose symptomatic intracerebral hemorrhage (sICH) within 24 h of IVT (9). The study received approval (LL-2021-LW-003, February 22, 2021) from the ethics committee at Yangpu Hospital of Tongji University School of Medicine. Patient information was kept confidential at Yangpu Hospital of Tongji University School of Medicine. The clinical studies were carried out in accordance with the guidelines of the Declaration of Helsinki.

### Baseline variable assessment

Patient records, encompassing admission data, were retrospectively analyzed to evaluate demographics, vascular risk factors, imaging, and laboratory data. A certified neurologist employed the NIHSS to assess baseline neurological deficits (10). Patient outcomes were gathered during clinic visits or telephone interviews conducted 3 months post-event. The main result was identified as poor functional status, indicated by a modified Rankin Scale (mRS) score between 3 and 6. Blood samples were taken from every patient prior to reperfusion therapy, and the GPR was computed by dividing the glucose concentration by the potassium concentration (11).

### Statistical analysis

Depending on the normality of their distribution, quantitative variables were expressed as mean ± standard deviation (SD) or median with interquartile range (IQR), whereas categorical variables were presented as frequencies and percentages. Continuous variables were analyzed using *t*-tests or Mann–Whitney U tests, while categorical variables were assessed with chi-square tests or Fisher’s exact tests. The 90-day adverse outcome odds ratio (OR) and 95% confidence interval (CI) for each unit rise in GPR and its quartiles were analyzed using logistic regression models. Adjustments were made to the crude model for variables that had a *p*-value below 0.05 in the univariate analysis, including age, sex, diabetes, smoking, history of stroke or transient ischemic attack (TIA), atrial fibrillation, sICH, baseline NIHSS score, proportion of mRS scores of 0–2 upon admission, blood pressure of systolic, C-reactive protein (CRP), hemoglobin (HB), platelet count (PLT), red blood cell count (RBC), glycated hemoglobin (HBA1c) and cholesterol associated with high-density lipoproteins (HDL-C), excluding glucose and potassium due to collinearity concerns. Model 1 was further adjusted for the crude model variables and additional factors such as hypertension, coronary artery disease, onset-to-needle time (ONT), blood pressure of diastolic, white blood cell count (WBC), creatinine (CRE), total cholesterol (TC), triglycerides (TG), cholesterol associated with low-density lipoprotein (LDL-C) and homocysteine (Hcy). Model 2 retained all the variables from Model 1, excluding the proportion of mRS 0–2 scores, and replaced it with baseline mRS as a continuous variable. Furthermore, to investigate the association between the GPR and prognosis in specific populations, we conducted a subgroup analysis based on identified risk factors, including gender, age, NIHSS score, history of stroke or transient ischemic attack, diabetes, atrial fibrillation, hypertension, and coronary artery disease. To determine the predictive value of GPR for a poor functional outcome at 90 days, receiver operating curve (ROC) analysis was utilized. Ultimately, to examine the dose–response relationship between the GPR and clinical functional outcome, restricted cubic splines (RCS) were applied, using three knots at the 10th, 50th, and 90th percentiles, while adjusting for covariates in model 2 (12).

Data processing and analysis were performed using R version 4.4.0 (2024-04-24), along with Zstats 1.0.^1^ Statistical significance was determined by a two-sided *p*-value below 0.05.

## Results

### Basic features

**Figure 1:**
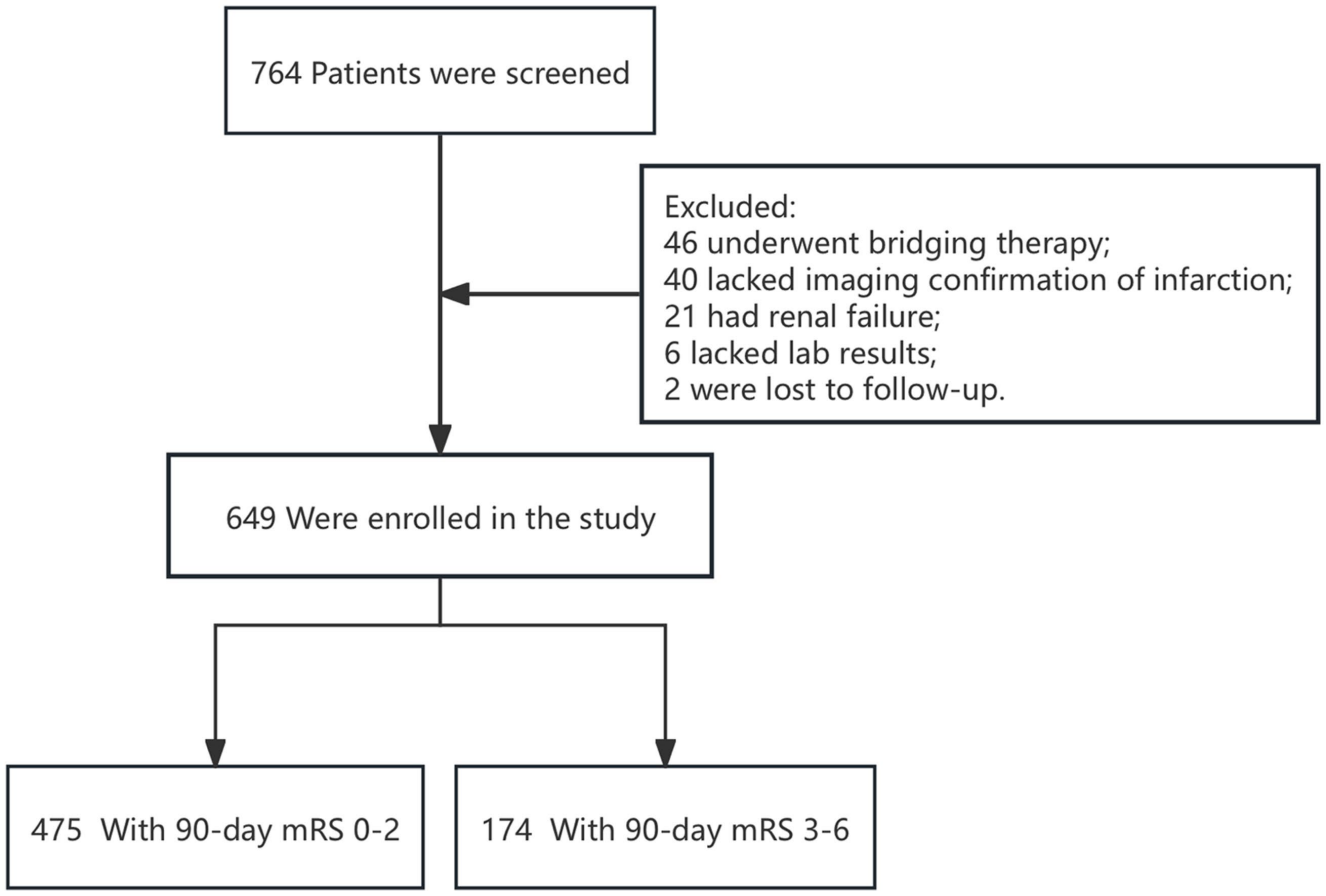
Research flow chart.

Throughout the study, 764 patients with acute ischemic stroke were treated with IVT. Of these patients, 46 underwent bridging therapy, 40 did not have imaging confirmation of infarction, 21 presented with renal failure, 6 had incomplete laboratory data, and 2 were not followed up on. These cases were subsequently excluded from the analysis (Figure 1). Consequently, data from 649 patients were included and analyzed. The enrolled subjects’ demographic details, laboratory information, and clinical characteristics are compiled in Table 1. Participants in the cohort had a median age of 71, with an interquartile range of 62 to 84 years, and consisted of 410 men. The initial median NIHSS score was 4, with an interquartile range of 2 to 8. Based on the Heidelberg Bleeding Classification, 17 patients (2.6%) were diagnosed with sICH. The median GPR levels among enrolled patients were 1.93 (IQR:1.58–2.47).

**Table 1:** Baseline information for patients experiencing both good and bad functional prognoses at 90 days.

Variables	Total population (*n* = 649)	Results after 90 days		*p*-value
		Favorable prognosis (mRS ≤ 2, *n* = 475)	Unfavorable prognosis (mRS > 3, *n* = 174)	
Age (years), median (IQR)	71 (62 ~ 84)	68 (61 ~ 80)	82 (70 ~ 88)	<0.001
Male, *n* (%)	410 (63.2)	323 (68.0)	87 (50.0)	<0.001
Risk factors, *n* (%)				
Smoking	285 (43.9)	230 (48.4)	55 (31.6)	<0.001
Diabetes	239 (36.8)	162 (34.1)	77 (44.3)	0.018
Stroke/TIA	148 (22.8)	94 (19.8)	54 (31.0)	0.002
Hypertension	471 (72.6)	340 (71.6)	131 (75.3)	0.348
Coronary artery disease	120 (18.5)	80 (16.8)	40 (23.0)	0.074
Atrial fibrillation	117 (18.0)	61 (12.8)	56 (32.2)	<0.001
Clinical data				
Time from onset-to-needle (min), median (IQR)	131 (125 ~ 137.5)	131 (122 ~ 135)	131 (131 ~ 142)	0.083
sICH, *n* (%)	17 (2.6)	6 (1.3)	11 (6.3)	<0.001
Systolic blood pressure (mmHg), median (IQR)	157 (142 ~ 173)	155 (141 ~ 171)	161 (144 ~ 183)	0.007
Diastolic blood pressure (mmHg), median (IQR)	86 (78 ~ 97)	86 (78 ~ 97)	86 (76 ~ 97)	0.624
NIHSS on admission, median (IQR)	4 (2 ~ 8)	4 (2 ~ 6)	9 (5 ~ 16)	<0.001
mRS 0–2 on admission, *n* (%)	637 (98.2)	475 (100.0)	162 (93.1)	<0.001
All-cause mortality at 90 days, *n* (%)	38 (5.8)	0 (0.0)	38 (21.8)	<0.001
Laboratory parameters, median (IQR)				
CRP, mg/L	5.00 (3.05 ~ 5.06)	5.00 (3.00 ~ 6.23)	5.00 (4.00 ~ 8.35)	0.007
WBC, 10^9^/L	7.10 (5.90 ~ 8.50)	7.10 (5.85 ~ 8.50)	7.30 (5.90 ~ 8.50)	0.976
RBC, 10^12^/L	4.60 (4.24 ~ 4.98)	4.63 (4.28 ~ 5.01)	4.52 (4.06 ~ 4.87)	0.002
PLT, 10^9^/L	206.00 (171.00 ~ 245.00)	212.00 (173.00 ~ 247.00)	195.50 (163.75 ~ 230.00)	0.013
HB, g/L	141.00 (129.00 ~ 152.00)	142.00 (131.00 ~ 153.00)	137.00 (121.00 ~ 149.00)	<0.001
HBA1c, %	6.00 (5.70 ~ 6.60)	6.00 (5.70 ~ 6.50)	6.25 (5.80 ~ 7.40)	<0.001
Glucose, mmol/L	7.25 (6.17 ~ 9.27)	7.05 (6.06 ~ 8.79)	7.88 (6.64 ~ 10.01)	<0.001
Potassium, mmol/L	3.87 (3.57 ~ 4.16)	3.88 (3.59 ~ 4.17)	3.77 (3.53 ~ 4.08)	0.031
GPR	1.93 (1.58 ~ 2.47)	1.88 (1.52 ~ 2.37)	2.14 (1.77 ~ 2.79)	<0.001
TG, mmol/L	1.18 (0.87 ~ 1.64)	1.24 (0.88 ~ 1.66)	1.13 (0.80 ~ 1.54)	0.056
TC, mmol/L	4.56 (3.83 ~ 5.37)	4.67 (3.83 ~ 5.39)	4.47 (3.78 ~ 5.27)	0.123
HDL-C, mmol/L	1.12 (0.95 ~ 1.33)	1.15 (0.96 ~ 1.34)	1.07 (0.90 ~ 1.27)	0.011
LDL-C, mmol/L	2.91 (2.36 ~ 3.46)	2.96 (2.39 ~ 3.49)	2.87 (2.33 ~ 3.40)	0.309
CRE, mmol/L	78.00 (65.00 ~ 95.00)	77.00 (65.00 ~ 95.00)	82.50 (67.00 ~ 93.25)	0.349
Hcy, mmol/L	14.82 (11.21 ~ 20.41)	14.94 (11.00 ~ 20.00)	15.45 (12.69 ~ 21.84)	0.071

TIA, transient ischemic attack; sICH, symptomatic intracerebral hemorrhage; NIHSS, National Institutes of Health stroke scale; CRP, C-reactive protein; WBC, white blood cell count; RBC, red blood cell count; PLT, platelet count; HB, hemoglobin; HBA1c, glycated hemoglobin; GPR, glucose-to-potassium ratio; TG, triglycerides; TC, total cholesterol; HDL-C, cholesterol associated with high-density lipoproteins; LDL-C, cholesterol associated with low-density lipoprotein; CRE, creatinine; Hcy, homocysteine.

### Correlation between GPR and functional outcomes

In the 90-day follow-up period, 174 patients (26.8%) had negative functional outcomes. Univariate analysis indicated that patients with unfavorable functional outcomes were significantly older than those without (median age: 82 years versus 68 years; *p* < 0.001). Patients with negative functional outcomes had significantly higher rates of diabetes (34.1% compared to 22.1%; *p* = 0.018), stroke or TIA (31.0% compared to 19.8%; *p* = 0.002), and atrial fibrillation (32.2% compared to 12.8%; *p* < 0.001). Additionally, patients with negative functional outcomes also had elevated initial NIHSS scores (median: 9 versus 4; *p* < 0.001), systolic blood pressure (median: 161 mmHg versus 155mHg; *p* = 0.006), HbA1c (median: 6.25% versus 6.00%; *p* < 0.001), CRP levels (median: 5.00 mg/L versus 5.00 mg/L; *p* = 0.007), and GPR (median: 2.14 versus 1.88; *p* < 0.001). They also had a higher incidence of sICH (6.3% versus 1.3%; *p* < 0.001) and all-cause mortality (21.8% versus 0%; *p* < 0.001). Conversely, they had lower proportions of smokers (31.6% versus 48.4%; *p* < 0.001), baseline mRS scores of 0–2 (100.0% versus 93.1%; *p* < 0.001), and male patients (50.0% versus 68.0%; *p* < 0.001). Furthermore, they showed lower levels of RBC (median: 4.52 versus 4.63; *p* = 0.002), PLT (median: 195.5 versus 212.00; *p* = 0.013), HB (median: 137.00 versus 142.00; *p* < 0.001), and HDL-c (median: 1.07 versus 1.15; *p* = 0.011; Table 1).

**Figure 2:**
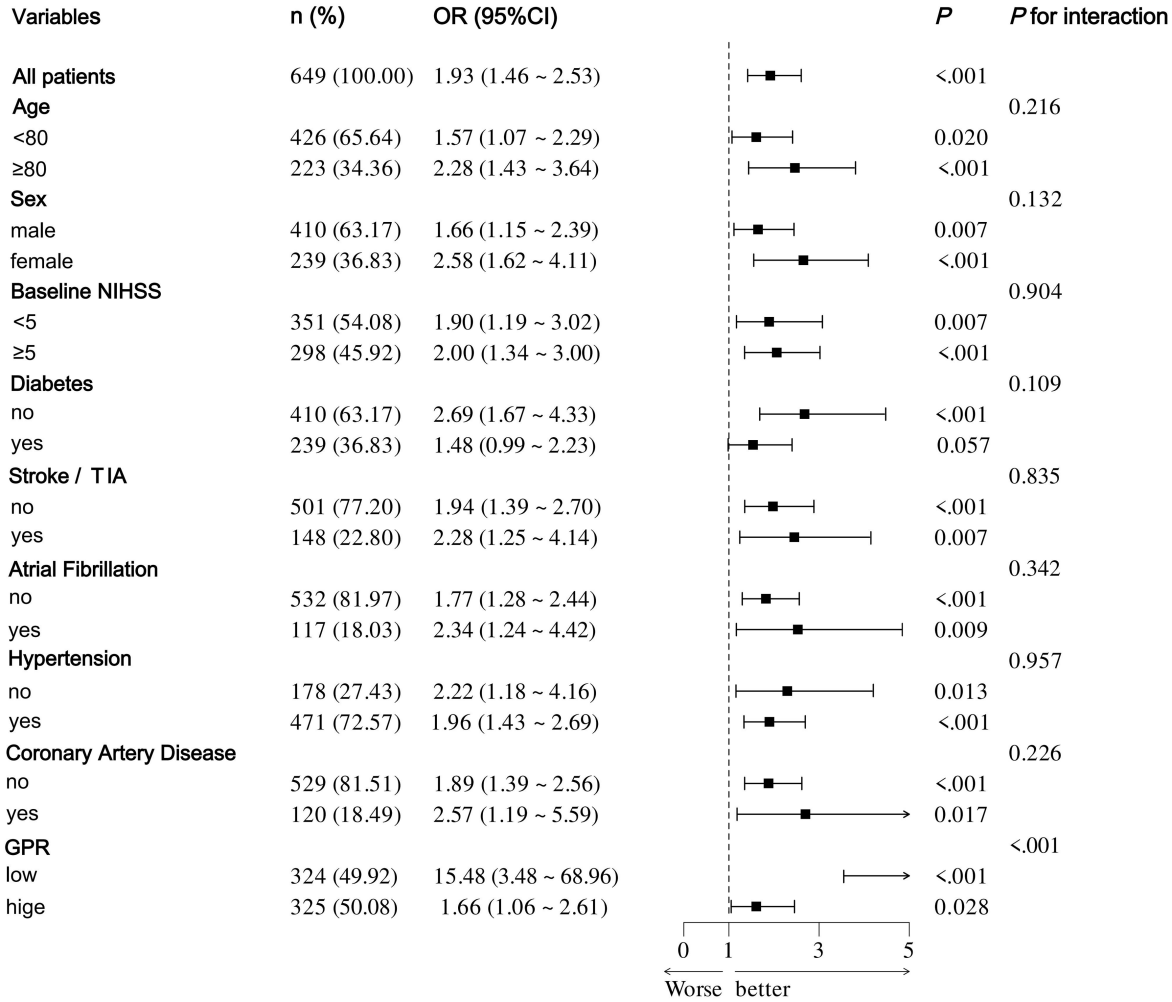
Forest plot of subgroup analysis by binary logistic regression of model 2.

**Figure 3:**
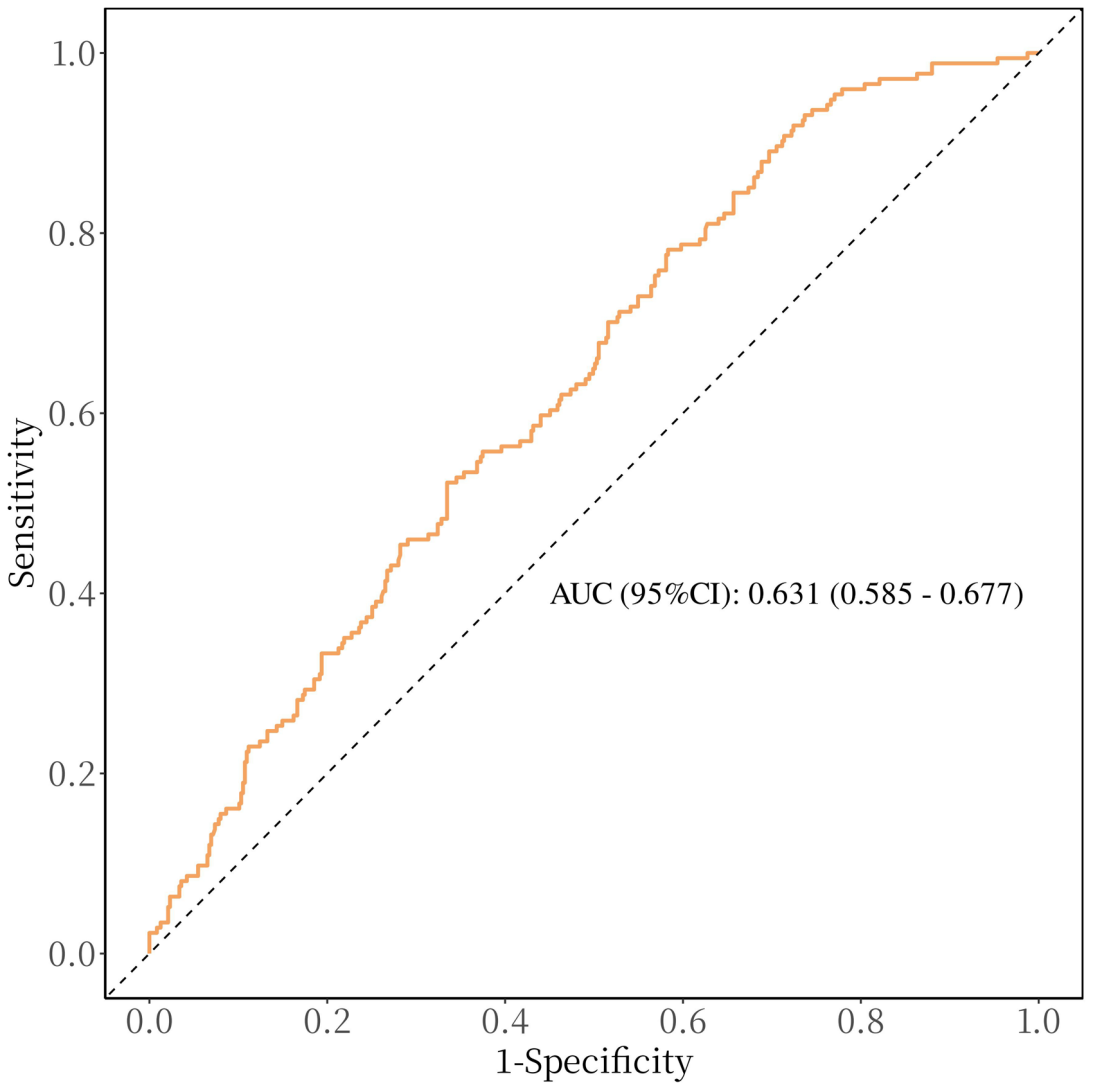
Receiver operating characteristic curve showing optimized cut-off for GPR in predicting poor functional outcomes (mRS 3–6). The optimal cutoff for predicting poor functional outcome is GPR ≥ 1.745 (sensitivity: 41.7%, specificity: 78.2%).

**Figure 4:**
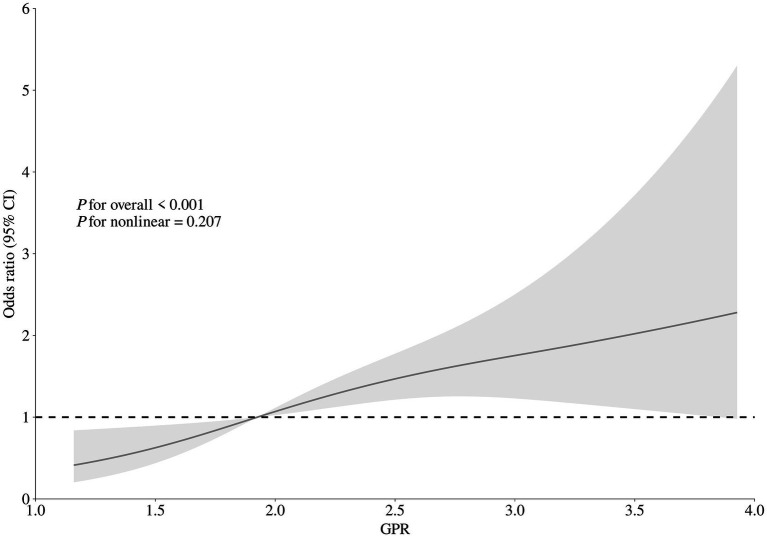
Correlation between GPR levels and risk of 90-day poor functional outcome. Odds ratio and 95% confidence intervals were derived from restricted cubic spline regression with three knots (at 10th, 50th, and 90th percentiles). The odds ratio was controlled for the same variables as model 2 in Table 2.

After accounting for possible confounding factors in multivariate logistic analysis, an increased GPR was notably linked to a greater likelihood of a negative functional outcome after 90 days (OR, 1.821; 95% CI, 1.340–2.473, *p* < 0.001). The detailed results of univariate and multivariate logistic regression analyses for the evaluation of functional prognostic factors are provided in Supplementary Table S1. Analyzing GPR in categorical terms yielded similar findings (Table 2). Simultaneously, utilizing model 2, to depict the findings of the subgroup multivariate logistic regression analysis, we developed a forest plot. The findings indicate that GPR exerts a more pronounced influence on the outcomes of non-diabetic patients in comparison to those with diabetes (Figure 2). Then, the results of the ROC analysis indicated that, across all patients, for predicting a poor functional prognosis at 90 days, the AUC of the GPR was 0.631 (95% CI, 0.585–0.677, *p* < 0.001). A cutoff value of 1.745 was identified as optimal, showing a sensitivity of 41.7% and a specificity of 78.2% (Figure 3). Additionally, the RCS analysis showed that GPR is positively linearly associated with unfavorable functional outcomes after 90 days (*p* = 0.207 for no linearity; *p* < 0.001 for overall; Figure 4).

**Table 2:** Univariate and multivariate logistic regression analysis for the relationship between GPR levels and 90-day poor functional outcome.

	Univariate regression		Crude model		Model 1		Model 2	
	OR (95% CI)	*p*-value	OR (95% CI)	*p*-value	OR (95% CI)	*p*-value	OR (95% CI)	*p*-value
GPR levels	1.806 (1.415–2.305)	<0.001	1.748 (1.300–2.352)	<0.001	1.951 (1.436–2.650)	<0.001	1.821 (1.340–2.473)	<0.001
GPR level quartiles								
1st	Reference	/	Reference	/	Reference	/	Reference	/
2nd	3.286 (1.832–5.894)	<0.001	2.082 (1.053–4.118)	0.035	1.980 (0.997–3.932)	0.051	2.235 (1.123–4.449)	0.022
3rd	3.097 (1.723–5.568)	<0.001	2.263 (1.153–4.441)	0.018	2.421 (1.229–4.769)	0.011	2.450 (1.236–4.858)	0.010
4th	4.341 (2.442–7.719)	<0.001	2.543 (1.245–5.196)	0.010	2.898 (1.416–5.934)	0.004	2.853 (1.378–5.906)	0.005

Quartiles of GPR level: 1st GPR <1.582; 2nd GPR = 1.582 ~ 1.925; 3rd GPR = 1.925 ~ 2.471; 4th GPR ≥2.471.

Crude model: variables with a *p*-value below 0.05 in the univariate analysis of baseline information, including age, sex, history of stroke or TIA, atrial fibrillation and diabetes, smoking, sICH, baseline NIHSS, proportion of mRS 0–2 on admission, blood pressure of systolic, CRP, HB, PLT, RBC, HBA1c, and HDL-C, excluding 90-day all-cause mortality as well as glucose and potassium due to collinearity concerns.

Model 1: adjusted for the crude model variables and additional factors such as hypertension, coronary artery disease, ONT, diastolic blood pressure, WBC, CRE, TC, TG, LDL-C, and Hcy.

Model 2: retained all the variables from Model 1, excluding the proportion of mRS 0–2 scores, and replaced it with baseline mRS as a continuous variable.

## Discussion

The research indicates a notable correlation between GPR and the functional results in AIS patients receiving IVT. Importantly, this correlation persisted even after taking into account potential confounding factors. Higher GPR levels upon admission were linked to an increased likelihood of negative functional outcomes 90 days after treatment. When GPR was analyzed as a categorical variable, there was an observed trend of increasing odds ratio values from the first to the fourth quartile. The impact of GPR on results was more significant in patients without diabetes than in those with the condition.

Our findings are consistent with previous research on various neurological disorders. For example, an earlier study discovered that the GPR upon admission could accurately forecast mortality rate within 30 days for patients suffering from ischemic stroke (6). Earlier research has emphasized the connection between GPR and unfavorable outcomes post-endovascular thrombectomy (5). Moreover, GPR has been proposed as a potential outcome marker for critical brain injuries and intracranial hemorrhage (13–15). The literature suggests that there is a close relationship between GPR and the Glasgow Coma Scale score, as well as cerebral vasospasm, in cases of aneurysmal subarachnoid hemorrhage (16, 17). Additionally, GPR has been analyzed as a forecast for the outcome of sudden intracerebral bleeding (13). This is also linked to prognosis in instances of severe traumatic brain injuries requiring surgical intervention, such as acute subdural hematoma, traumatic subarachnoid hemorrhage, acute epidural hematoma, and brain contusion due to trauma (18). In this study, patients with ischemic stroke who received IVT and had a greater GPR at admission was associated with a greater likelihood of a poor functional outcome after 90 days, especially in non-diabetic individuals. However, the basic pathological mechanism remains incompletely explained.

The prognostic value of GPR builds upon previous findings regarding the individual roles of glucose and potassium in stroke outcomes. Post-stroke hyperglycemia is triggered by increased cortisol and catecholamine levels after an ischemic injury, associated with increased infarct size, oxidative stress, and disruption of the blood–brain barrier (7, 19). Early stroke mortality was independently linked to hyperglycemia, irrespective of diabetes status (20). Earlier research identified the connection between stress hyperglycemia ratio and patients’ clinical outcomes over 90 days, undergoing EVT for stroke caused by an acute blockage in a large vessel (19, 21). The results of this study are consistent with the latest guidelines issued by the European Stroke Organization (ESO) concerning intravenous thrombolysis for acute ischemic stroke. These guidelines underscore the importance of actively managing hyperglycemia with insulin in patients undergoing intravenous thrombolysis for acute ischemic stroke. Such management is crucial for optimizing therapeutic outcomes and enhancing patient prognosis (22). In the same way, serum potassium is vital for keeping the body’s internal environment stable and lowering the risk of stroke (23, 24). Hypokalemia has been linked to impaired neuronal repair, increased excitotoxicity, and heightened risk of recurrent stroke. According to Johnson et al., there is a linear relationship between potassium levels in the blood during early middle age and the potential for ischemic stroke, brain bleeding, and overall mortality (25). A study from China showed that decreased levels of serum potassium are connected to the risk of recurrent AIS or TIA (26).

The stress-related hypothalamic–pituitary–adrenal (HPA) axis and the renin-angiotensin-aldosterone system (RAAS) activation is reflected in high blood sugar and low potassium levels. Previous studies have demonstrated that the GPR serves as a marker for stress activity and RAAS response. A physiological mechanism through which GPR forecasts the 90-day results for patients who suffered an ischemic stroke after IVT includes the joint activation of the HPA axis and RAAS in reaction to stress. This interaction leads to overproduction of catecholamines, which increases serum glucose levels, thereby stimulating insulin secretion and facilitating the intracellular transport of serum potassium (27, 28). Consequently, by integrating these two parameters, it captures the synergistic effects of metabolic and ionic imbalance: the higher the GPR, the more severe the physiological damage.

While we found a connection between GPR and functional outcome after 90 days, some restrictions should be acknowledged. Firstly, there is a possibility of selection bias and ongoing confounding due to the study’s retrospective design. Secondly, GPR was measured only at admission, and serial measurements might better capture the dynamic changes in metabolic and ionic states during stroke progression. Finally, the study was performed in one place, potentially limiting the relevance of the outcomes. In conclusion, our study identifies serum GPR as a significant prognostic biomarker for poor functional outcomes in AIS patients treated with IVT. These results indicate that assessing GPR could be a useful parameter for tracking outcomes after IVT. Broader studies are necessary to fully understand these relationships, potentially assisting in identifying patients at high risk for adverse outcomes post-IVT and stressing the importance of glucose and potassium.

## Data Availability

The raw data supporting the conclusions of this article will be made available by the authors, without undue reservation.

## Footnotes

^1^ www.zstats.net
