# Supplementary material for: The glucose-to-potassium ratio: a predictor of poor functional outcomes in stroke patients receiving thrombolytic therapy
Source: Front Neurol. 2025 Jun 5;16:1581747. doi: 10.3389/fneur.2025.1581747 (PMC12177222; doi:10.3389/fneur.2025.1581747)
Supplement: Supplementary file 1 [file Table_1.DOCX]

Supplementary Table S1. Univariate and Multivariate Logistic Regression Analysis Results for Functional Prognostic Factors.

| **Variables** | **Univariate logistic regression** | | | | | **Multivariate logistic regression** | | | | |
| --- | --- | --- | --- | --- | --- | --- | --- | --- | --- | --- |
|  | β | S.E | Z | P | OR (95%CI) | β | S.E | Z | P | OR (95%CI) |
| Sex |  |  |  |  |  |  |  |  |  |  |
| Male |  |  |  |  | Reference |  |  |  |  |  |
| Female | 0.754 | 0.181 | 4.171 | <.001 | 2.125 (1.491 ~ 3.028) |  |  |  |  |  |
| sICH |  |  |  |  |  |  |  |  |  |  |
| No |  |  |  |  | Reference |  |  |  |  | Reference |
| Yes | 1.663 | 0.516 | 3.225 | 0.001 | 5.275 (1.920 ~ 14.491) | 1.390 | 0.599 | 2.321 | 0.020 | 4.017 (1.242 ~ 12.993) |
| Stroke/TIA |  |  |  |  |  |  |  |  |  |  |
| No |  |  |  |  | Reference |  |  |  |  |  |
| Yse | 0.601 | 0.200 | 3.001 | 0.003 | 1.824 (1.232 ~ 2.701) |  |  |  |  |  |
| Hypertension |  |  |  |  |  |  |  |  |  |  |
| No |  |  |  |  | Reference |  |  |  |  |  |
| Yes | 0.190 | 0.203 | 0.937 | 0.349 | 1.210 (0.812 ~ 1.801) |  |  |  |  |  |
| Atrial Fibrillation |  |  |  |  |  |  |  |  |  |  |
| No |  |  |  |  | Reference |  |  |  |  | Reference |
| Yes | 1.170 | 0.212 | 5.505 | <.001 | 3.221 (2.124 ~ 4.885) | 0.449 | 0.270 | 1.660 | 0.097 | 1.567 (0.922 ~ 2.662) |
| Coronary Artery Disease |  |  |  |  |  |  |  |  |  |  |
| No |  |  |  |  | Reference |  |  |  |  |  |
| Yes | 0.388 | 0.218 | 1.780 | 0.075 | 1.474 (0.962 ~ 2.259) |  |  |  |  |  |
| Smoking |  |  |  |  |  |  |  |  |  |  |
| No |  |  |  |  | Reference |  |  |  |  |  |
| Yes | -0.709 | 0.187 | -3.787 | <.001 | 0.492 (0.341 ~ 0.710) |  |  |  |  |  |
| Diabetes |  |  |  |  |  |  |  |  |  |  |
| No |  |  |  |  | Reference |  |  |  |  |  |
| Yes | 0.428 | 0.181 | 2.366 | 0.018 | 1.534 (1.076 ~ 2.186) |  |  |  |  |  |
| Age | 0.065 | 0.008 | 7.763 | <.001 | 1.067 (1.050 ~ 1.085) | 0.035 | 0.011 | 3.185 | 0.001 | 1.036 (1.014 ~ 1.059) |
| Systolic blood pressure | 0.009 | 0.003 | 2.597 | 0.009 | 1.009 (1.002 ~ 1.016) | 0.010 | 0.004 | 2.435 | 0.015 | 1.010 (1.002 ~ 1.019) |
| Diastolic blood pressure | -0.001 | 0.006 | -0.212 | 0.832 | 0.999 (0.987 ~ 1.011) |  |  |  |  |  |
| Time from onset-to-needle | 0.003 | 0.002 | 1.194 | 0.233 | 1.003 (0.998 ~ 1.007) |  |  |  |  |  |
| mRS on admission | 0.999 | 0.215 | 4.639 | <.001 | 2.716 (1.781 ~ 4.142) | 0.868 | 0.246 | 3.531 | <.001 | 2.381 (1.471 ~ 3.855) |
| NIHSS on admission | 0.168 | 0.018 | 9.517 | <.001 | 1.183 (1.143 ~ 1.225) | 0.144 | 0.020 | 7.327 | <.001 | 1.155 (1.111 ~ 1.200) |
| CRP | 0.015 | 0.006 | 2.594 | 0.009 | 1.015 (1.004 ~ 1.027) |  |  |  |  |  |
| WBC | 0.002 | 0.038 | 0.040 | 0.968 | 1.002 (0.929 ~ 1.079) |  |  |  |  |  |
| RBC | -0.441 | 0.156 | -2.834 | 0.005 | 0.643 (0.474 ~ 0.873) | 0.592 | 0.358 | 1.654 | 0.098 | 1.808 (0.896 ~ 3.646) |
| PLT | -0.004 | 0.002 | -2.343 | 0.019 | 0.996 (0.994 ~ 0.999) | -0.003 | 0.002 | -1.457 | 0.145 | 0.997 (0.993 ~ 1.001) |
| HB | -0.020 | 0.005 | -4.020 | <.001 | 0.980 (0.971 ~ 0.990) | -0.019 | 0.011 | -1.663 | 0.096 | 0.981 (0.959 ~ 1.003) |
| HBA1c | 0.199 | 0.065 | 3.051 | 0.002 | 1.220 (1.074 ~ 1.386) |  |  |  |  |  |
| GPR | 0.591 | 0.125 | 4.747 | <.001 | 1.806 (1.415 ~ 2.305) | 0.599 | 0.156 | 3.834 | <.001 | 1.821 (1.340 ~ 2.473) |
| TG | -0.176 | 0.123 | -1.434 | 0.152 | 0.839 (0.660 ~ 1.067) | 0.290 | 0.168 | -1.729 | 0.084 | 0.748 (0.539 ~ 1.039) |
| TC | -0.108 | 0.078 | -1.374 | 0.169 | 0.898 (0.770 ~ 1.047) |  |  |  |  |  |
| HDL-C | -0.726 | 0.329 | -2.210 | 0.027 | 0.484 (0.254 ~ 0.921) | -1.451 | 0.459 | -3.160 | 0.002 | 0.234 (0.095 ~ 0.576) |
| LDL-C | -0.086 | 0.105 | -0.820 | 0.412 | 0.918 (0.747 ~ 1.127) | 0.295 | 0.143 | 2.066 | 0.039 | 1.344 (1.015 ~ 1.778) |
| CRE | 0.000 | 0.001 | 0.326 | 0.744 | 1.000 (0.998 ~ 1.003) |  |  |  |  |  |
| Hcy | 0.010 | 0.008 | 1.317 | 0.188 | 1.010 (0.995 ~ 1.026) |  |  |  |  |  |

TIA, transient ischemic attack; sICH, symptomatic intracerebral hemorrhage; NIHSS, National Institutes of Health stroke scale; CRP, C-reactive protein; WBC, white blood cell count; RBC, red blood cell count; PLT, platelet count; HB, hemoglobin; HBA1c, glycated hemoglobin; GPR, glucose-to-potassium ratio; TG, triglycerides; TC, total cholesterol; HDL-C, cholesterol associated with high-density lipoproteins; LDL-C, cholesterol associated with low-density lipoprotein; CRE, creatinine; Hcy, homocysteine.
